# Polyether- and Tertiary Amine-Modified Silicone Surfactants: Synthesis and Surface Performance Across pH Ranges

**DOI:** 10.3390/polym17091204

**Published:** 2025-04-28

**Authors:** Yi Guo, Cheng Yao

**Affiliations:** 1 School of Chemistry & Molecular Engineering, Nanjing Tech University, No. 30 Puzhunan Road, PuKou District, Nanjing 211816, China; ike_guo@njtech.edu.cn; 2 Jiangsu OSiC New Materials Technology Research Co., Ltd., No. 56 Nanhai Road, Zhangjiagang Chemical Industrial Park, Suzhou 215633, China

**Keywords:** polyether- and tertiary amine-modified silicone surfactants, static and dynamic surface tension, critical micelle concentration, surface activities, thermodynamic parameters of adsorption and micellization

## Abstract

In this study, polymerized silicone surfactants were modified with polyether and tertiary amine groups with the aim of improving the surface performance. Various PSiEO/(PO)-OH(CH_3_) surfactants were synthesized and their structures and performance were characterized through ^1^H NMR, FTIR spectroscopy, static surface tension, dynamic surface tension, zeta potential, and dynamic light scattering measurements. Subsequently, the modified silicones were incorporated as surfactants in aqueous solutions with different pH values. The surfactants with different hydrophobic/hydrophilic groups and end-capping groups exhibited different surface performances over a wide pH range. Thermodynamic parameters indicated that the micellization and adsorption of these surfactants were endothermic and spontaneous processes driven by entropy. The processes were hindered by increasing the solution pH and modification with hydrophobic groups. The aggregation behavior was significantly different under acidic, neutral, and basic aqueous conditions.

## 1. Introduction

Polymer surfactants have many commercial applications [1,2,3] such as stabilizing emulsions in the food and chemical industries, stabilizing foams in the polyurethane industry, and adjusting the surface tension in coating processes to enhance the surface and interfacial properties [4,5]. They are also prevalent in many biological systems, where their ability to modulate membrane fluidity and protein interactions underscores their biological relevance [6]. Therefore, investigating the adsorption behavior of polymer surfactants is crucial [7,8,9]. While extensive studies have focused on linear or single-functional surfactants, recent advances highlight the need for multifunctional systems capable of adapting to dynamic environmental conditions, such as variable pH [10,11]. Many researchers have studied the surface adsorption behavior of polymer surfactants from the perspective of the structure of macromolecular surfactants at the gas–liquid interface. However, the dynamic and static surface tensions also offer valuable insights into the adsorption and formation behavior of colloidal surfactant particles at the interface [12,13]. Notably, the interplay between surfactant architecture (e.g., branched chains, mixed hydrophilic/hydrophobic segments) and interfacial dynamics remains underexplored, particularly in systems requiring pH responsiveness [14]. Several reports on the static and dynamic surface tensions of polymer surfactants have recently been published [9,15].

Polyether-modified silicone copolymers are amphiphilic molecules with similar interfacial properties to surfactants. The polyethylene oxide block in these polymers is hydrophilic, whereas the polypropylene oxide block is hydrophobic. This amphiphilicity means that these polymers form micelles at gas–liquid interfaces owing to adsorption behavior. The surface properties are also influenced by the terminating groups of the polyether segments [16,17]. For example, polyether-modified silicone surfactants are nonionic, whereas the introduction of cationic groups makes the surfactant ionic. This increases their potential for use in pH-responsive, antistatic, antibacterial, and catalytic applications [18]. Despite these advances, existing systems often face trade-offs between pH adaptability and interfacial efficiency. For instance, purely polyether-based surfactants exhibit limited responsiveness to pH changes, while cationic modifications may compromise colloidal stability under alkaline conditions [19]. To address these limitations, recent efforts have explored hybrid designs combining multiple functional groups, yet systematic studies on silicone surfactants with dual polyether and tertiary amine modifications are scarce [20].

In this study, a series of polyether- and tertiary amine-modified polysiloxane surfactants (denoted as PSiEO/(PO)-OH(CH_3_)) were designed, and their structures were characterized. Their surface performances were then investigated using dynamic and static surface tension measurements. To further study the properties of the surfactants, we evaluated their thermodynamic parameters and aggregation behavior in acidic, neutral, and basic environments. Unlike previous single-functional systems, our dual-functional design integrated polyether chains and tertiary amine groups to synergistically control micellization and interfacial dynamics across a broad pH range (3–11). Structural optimization through hydrophobic PO segments and acetyl capping enhanced molecular packing and aggregation behavior, bridging stimuli-responsiveness with practical performance. This approach advances pH-adaptive surfactants for applications such as smart coatings and drug delivery systems.

## 2. Materials and Methods

### 2.1. Materials

Octamethylcyclotetrasiloxane (D_4_, >99.5%; Dowcorning, Midland, MI, USA), polymethylhydrosiloxane (high-SiH silicone oil, viscosity 20 mm^2^ s^−1^, *M*_w_ = 2000; Dowcorning, Midland, MI, USA), hexamethyldisiloxane (HMDSO, >99.5%; Wacker Chemie AG, Burghausen, Bavaria, Germany), allyl polyethylene ether glycol (APEG, *M*_w_ = 600; Zhongshan Chemie, Nanjing, China), acetyl end-capped allyl polyethylene ether glycol (AAPEG, *M*_w_ = 600; Zhongshan Chemie, Nanjing, China), allyl polyethylene-polypropylene ether glycol (APEPG, *M*_w_ = 600, polyethylene/polypropylene = 1:1 *w*/*w*; Zhongshan Chemie, Nanjing, China), acetyl end-capped allyl polyethylene-polypropylene ether glycol (AAPEPG, *M*_w_ = 600, polyethylene/polypropylene = 1:1 *w*/*w*; Zhongshan Chemie, Nanjing, China), diethylamine (DEA, >99.0%; Aladdin Biochemical Technology, Shanghai, China), potassium hydroxide (KOH, >99.0%; Aladdin Biochemical Technology, Shanghai, China), sodium carbonate (Na_2_CO_3_, >98.0%; Aladdin Biochemical Technology, Shanghai, China), chloroplatinic acid (>39.0 wt.% Pt; Johnson Matthey PLC, Royston, Hertfordshire, UK), sulfuric acid (>98.0%; Aladdin Biochemical Technology, Shanghai, China), allyl glycidyl ether (AGE, >99.5%; Aladdin Biochemical Technology, Shanghai, China), isopropanol (IPA, >99%; Aladdin Biochemical Technology, Shanghai, China), and xylene (>99.5%; Aladdin Biochemical Technology, Shanghai, China) were used in the experiments. All reagents were of analytical grade. The solvent was distilled before use in all experiments.

### 2.2. Synthesis of Polyether- and Tertiary Amine-Modified Silicone Surfactants

**Figure 1:**
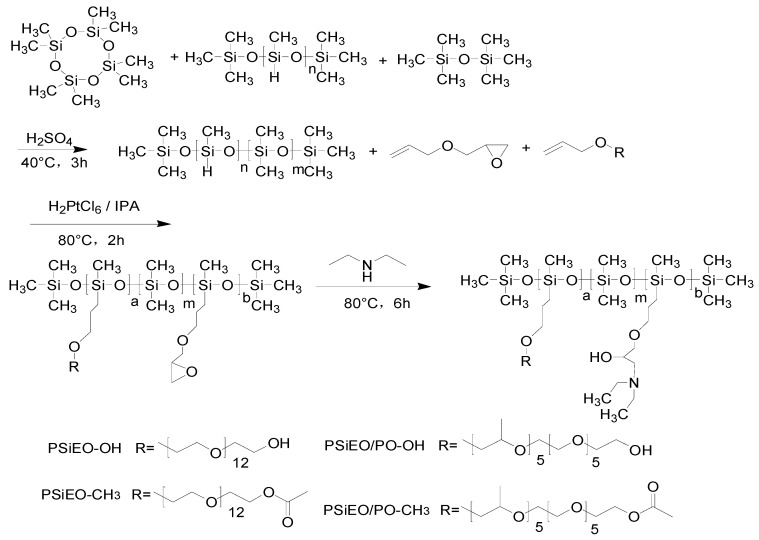
Synthesis of polyether- and tertiary amine-modified silicone surfactants.

Four polyether- and tertiary amine-modified silicone surfactants were prepared using different allyl polyethers (APEG, AAPEG, APEPG, and AAPEPG). The synthesis followed a three-step process, as shown in Figure 1, which involved the synthesis of low-hydrogen silicone oil, the preparation of polyether- and epoxy-modified polysiloxanes, and, finally, the preparation of polyether- and tertiary amine-modified silicone surfactants. The steps are described in Section 2.2.1, Section 2.2.2, and Section 2.2.3, respectively.

#### 2.2.1. Synthesis of Low-Hydrogen Silicone Oil

High-SiH silicone oil (52 g), D_4_ (238 g), HMDSO (10 g), and sulfuric acid (4 g) were mixed and heated at 40 ± 1 °C for 3 h. The sulfuric acid acted as a catalyst for the equilibration reaction between D4 and PMHS (polymethylhydrosiloxane), redistributing Si-H groups along the siloxane backbone. Excess Na_2_CO_3_ was added to neutralize the sulfuric acid. The neutralization reaction lasted 2 h, with stirring, to ensure complete salt precipitation and excess Na_2_CO_3_ and Na_2_SO_4_ produced by neutralization were filtered out. Then, the product was pumped, under vacuum, at 140 °C to remove all unreacted materials, yielding low-hydrogen silicone oil.

#### 2.2.2. Preparation of Polyether- and Epoxy-Modified Polysiloxane

The low-hydrogen silicone oil (200 g) and allyl polyether (APEG, AAPEG, APEPG, or AAPEPG; 60 g) were added to xylene (100 g) as the reaction solvent along with chloroplatinic acid (0.1 g) as a catalyst. The hydrosilylation reaction between SiH and allyl groups was conducted at 80 ± 1 °C for 2 h under nitrogen protection. Subsequently, AGE (60 g) was added to the mixture, and another 0.1 g of chloroplatinic acid was added. The mixture was reacted for another 1 h to graft epoxy groups onto the polysiloxane backbone. Reaction progress was monitored by FTIR spectroscopy (Thermo Scientific Nicolet iS20, Madison, WI, USA) where the disappearance of the SiH absorption peak at 2160 cm^−1^ confirmed completion. The reactant was then heated to 140 ± 1 °C and pumped, under vacuum, to remove the xylene and unreacted AGE, yielding polyether- and epoxy-modified polysiloxane. The epoxy content was determined to be 0.25 mmol·g^−1^ by HCl–acetone titration.

#### 2.2.3. Preparation of Polyether- and Tertiary Amine-Modified Silicone Surfactants

The polyether- and epoxy-modified polysiloxane (200 g) and DEA (40 g) were added to a reactor. The ring-opening reaction between epoxy groups and DEA was carried out at 80 ± 1 °C for 6 h with mechanical stirring (300 rpm). The reaction mechanism involved nucleophilic attack of the amine on the epoxy ring, forming a tertiary amine and hydroxyl group. Unreacted DEA was removed by vacuum stripping at 90 °C for 2 h. The product was purified by celite filtration to eliminate residual catalyst and byproducts. The polyether- and tertiary amine-modified silicone surfactants produced using APEG, AAPEG, APEPG, and AAPEPG were denoted as PSiEO-OH, PSiEO-CH_3_, PSiEO/PO-OH, and PSiEO/PO-CH_3_, respectively (and collectively as PSiEO/(PO)-OH(CH_3_)). The PSiEO/(PO)-OH(CH_3_) products were dried under vacuum at 60 °C for 24 h for further analysis.

### 2.3. Structural Characterization

The structures of the polyether- and tertiary amine-modified silicone surfactants were analyzed by FTIR spectroscopy (Thermo Scientific Nicolet iS20, Madison, WI, USA) between 400 and 4000 cm^−1^. Typical peaks were observed at 2870, 2959 (*ν*C–H, Si–CH_3_), and 1402 (*ν*C–H, –CH_2_), and 1260 (*ν*C–H, Si–CH_3_), 1010 (*ν*Si–O–Si), and 3429 cm^−1^ (*ν*N–H). The surfactants prepared using acetyl end-capped allyl polyethers (PSiEO-CH_3_ and PSiEO/PO-CH_3_) also produced another peak at 1739 cm^−1^ (*ν*C=O). ^1^H NMR was performed using a 400 MHz spectrometer (Avance III HD, Bruker, BioSpin GmbH, Ettlingen, Germany). The results are given in Supplementary Materials Figures S1–S4.

### 2.4. ^1^H NMR Spectroscopy at Different pH Values

To observe the effects of quaternization, ^1^H NMR spectroscopy was conducted at different pH values using a 400 MHz spectrometer (Avance III HD, Bruker, BioSpin GmbH, Ettlingen, Germany) with deuterium oxide (D_2_O) as the solvent and tetramethylsilane as the internal standard. The pH values of the PSiEO/(PO)-OH(CH_3_) surfactant solutions were adjusted using deuterated hydrochloric acid (DCl) and deuterated sodium hydroxide (NaOD).

### 2.5. Determination of Acid-Ionization Constant (pK_a_)

The acid-ionization constants (p*K*_a_) of the PSiEO/(PO)-OH(CH_3_) surfactants were determined by potentiometric titration. The surfactant was diluted to a mass concentration of 2 g L^−1^; then, the dilute solution (80 mL) was placed in a 100 mL beaker and the pH was adjusted to approximately 2.0 using 0.10 mol L^−1^ HCl. A magnetic stirrer was added to the beaker and placed on the stirring device (80 rpm). The solution was titrated with 0.10 mol L^−1^ NaOH at a constant temperature of 25 °C until the solution pH reached 12.0. The pH value was recorded after each titration.

### 2.6. Static Surface Tension

The static surface tensions of the solutions were examined using the platinum ring method at 25 ± 0.1 °C using a BYZ-1 automatic surface tension meter (Shanghai Sunny Hengping Scientific Instrument, Shanghai, China). The solutions were prepared with triple-distilled water, and the measurements were repeated until the results were reproducible. The data used were the averages of the actual measured data. To avoid contamination, the ring was washed and burned until reddish between measurements.

### 2.7. Dynamic Surface Tension

The dynamic surface tension was measured at 25 ± 0.2 °C using a SITA science line t100 tensiometer (Messtechnik GmbH, Darmstadt, Germany). A concentration of 2 g L^−1^ was used for all samples, and the solutions were prepared with triple-distilled water. The capillary was rinsed with ethanol three times and distilled water twice between measurements.

### 2.8. Zeta Potential and Dynamic Light Scattering (DLS)

Aqueous PSiEO/(PO)-OH(CH_3_) solutions with different pH values were prepared and ultrasonically dispersed for 30 min. The zeta potential and particle size were measured using a Nano-ZS dynamic laser light scattering instrument (Masterizer-2000, Malvern, Westborough, MA, UK).

## 3. Results and Discussion

### 3.1. Acid-Ionization Constant (pK_a_) of PSiEO/(PO)-OH(CH_3_) Surfactants

**Figure 2:**
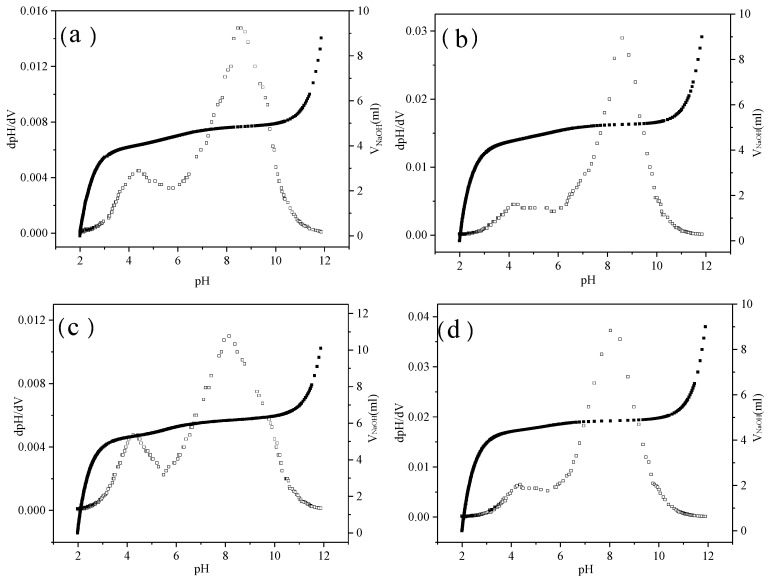
pH vs. *V*_NaOH_ (solid squares) and pH vs. dpH/d*V* (open squares) curves of (**a**) PSiEO-OH, (**b**) PSiEO-CH_3_, (**c**) PSiEO/PO-OH, and (**d**) PSiEO/PO-CH_3_.

The amino group in amino-modified polyether silicones is a weak base, and the lone pair of electrons on the N atom can accept free H^+^ in solution, forming a cationic surfactant. The acid-ionization constants (p*K*_a_) of the PSiEO/(PO)-OH(CH_3_) surfactants were measured by acid–base titration to obtain the distribution of different protonated states. Figure 2 shows the pH titration curves (pH vs. volume of NaOH (*V*_NaOH_) and pH vs. change in pH/change in volume of NaOH (dpH/d*V*)) of the surfactant solutions at a concentration of 5 g L^−1^. The peaks of the pH vs. dpH/d*V* curves correspond to the p*K*_a_ values.

All four PSiEO/(PO)-OH(CH_3_) surfactants exhibited two apparent titration jump points with the gradual addition of NaOH solution. The first jump appeared at pH 3.0, while the second jump commenced at pH 6.0. The first jump corresponded to the deprotonation of the amino-modified polyether silicone surfactant at pH 3.0. The second jump corresponded to the release of the wrapped tertiary amino group, because the amino-modified polyether silicone surfactant molecule gradually expanded as NaOH was continuously titrated into the solution. Thus, these jumps represent a transition from cationic to cationic/nonionic to nonionic. The p*K*_a_ values of the PSiEO/(PO)-OH(CH_3_) surfactants, as obtained from the acid–base titration curves, are listed in Table 1.

To determine the specific state of the four PSiEO/(PO)-OH(CH3) surfactants at different solution pH values, the zeta potential was measured under different pH conditions (surfactant concentration: 1 g L^−1^). The zeta potential indicates the stability of colloidal dispersion systems [21]. When the absolute value of the zeta potential is high, particles show mutual repulsion, and the dispersion is stable. When the absolute value of the zeta potential is low, particles show mutual attraction, resulting in aggregation or agglomeration; thus, the dispersion is unstable. Typically, dispersions with zeta potentials between 30 and −30 mV are unstable, whereas those with zeta potentials greater than 30 mV or less than −30 mV are stable [22].

**Figure 3:**
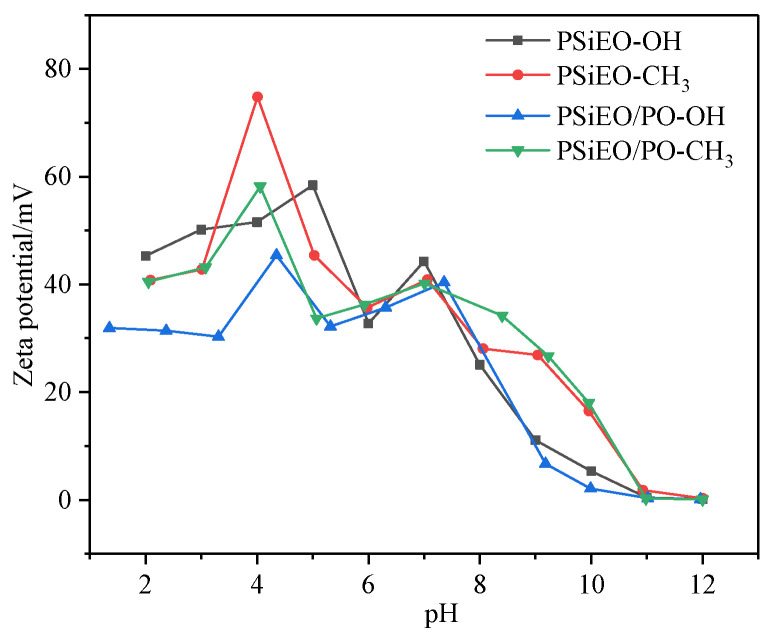
Zeta potential of PSiEO/(PO)-OH(CH_3_) surfactants at different pH values.

The zeta potentials of the four surfactants are shown in Figure 3 as a function of pH. Two peaks were observed at pH values of approximately 4 and 7. The first peak (pH 4) corresponded to the quaternization of some tertiary amino groups. The adsorption capacity of the solution interface gradually saturated with increasing pH, and minimum surface absorption area (*A*_min_) of the micelles at the surface also increased. However, the size of the particles decreased. When the interface could not accommodate all the surfactant, the morphology of the surfactant particles began to change, leading to the formation of larger micelles or other forms of micelles.

As more surfactant molecules aggregated together, thereby increasing the micelle size, some of the tertiary amine groups were situated in the interior of the micelle; these groups could not be combined with further counter ions to remove H^+^. At approximately pH 7, the quaternary ammonium salts inside the micelle began to deprotonate, causing a second peak. The zeta potentials of the PSiEO/(PO)-OH(CH_3_) surfactants decreased continuously as further NaOH was added. At pH 8–9, the zeta potentials decreased below 30 mV, indicating that the micelle systems were unstable owing to the continuous removal of H^+^ by surfactant molecules. The solution transitioned into a nonionic state, and the repulsive force between the molecules was small, resulting in instability of the system. At pH 11, the zeta potentials were nearly zero, and adding more NaOH did not change the zeta potential of the solution further. Although the zeta potentials do not provide accurate solution pH corresponding to the jump point, these results verify the two p*K*_a_ phenomena observed during acid–base titration.

### 3.2. ^1^H NMR Analysis at Different pH Values

To further verify the molecular form of the surfactant at different solution pH values, we evaluated the ^1^H NMR spectra of deuterated aqueous solutions of the PSiEO/PO-OH surfactant at pH 11.0, 7.0, and 3.0 (Figure S5). The chemical shifts differed with pH, indicating that PSiEO/PO-OH exists in different molecular forms at different solution pH values. With increasing pH, the chemical shifts of H^+^ positioning two or more C atoms further from the N atom remained largely constant, whereas the chemical shifts of H^+^ (h, g) closer to the N atom gradually disappeared. This indicates that PSiEO/PO-OH underwent deprotonation when the solution changed from acidic to basic, which further confirms the results of the acid–base titration and zeta potential measurements; that is, deprotonation still occurred when the pH was greater than 7. Furthermore, the ^1^H NMR spectrum of PSiEO/PO-OH was the same in D_2_O/NaOD at pH 11.0 and in deuterated chloroform (CDCl_3_) (Figure S6), indicating that the PSiEO/PO-OH molecules were nonionic at pH 11.0.

### 3.3. Static Surface Tension of PSiEO/(PO)-OH(CH_3_) Surfactants

Static surface tension measurements were used to investigate the adsorption behavior of the PSiEO/(PO)-OH(CH_3_) surfactants. Their physicochemical properties were compared by using the Gibbs equation (Equation (1)) [23]:

(1) $$\Gamma_{max}=-\frac{1}{2.303nRT}\times {\left[\frac{d\gamma }{dlogC\;}\right]}_{T}$$

where *Γ_max_* is the maximum surface excess concentration, *n* is a constant related to the structure of the surfactant, *R* is the universal gas constant (8.314 J mol^−1^ K^−1^), *T* is the absolute temperature (298.15 K), *γ* is the surface tension, and *C* is the surfactant concentration. *n* = 2 for a 1:1 ionic surfactant and *n* = 3 for other surfactants [24]. Therefore, in our study, *n* = 3.

**Figure 4:**
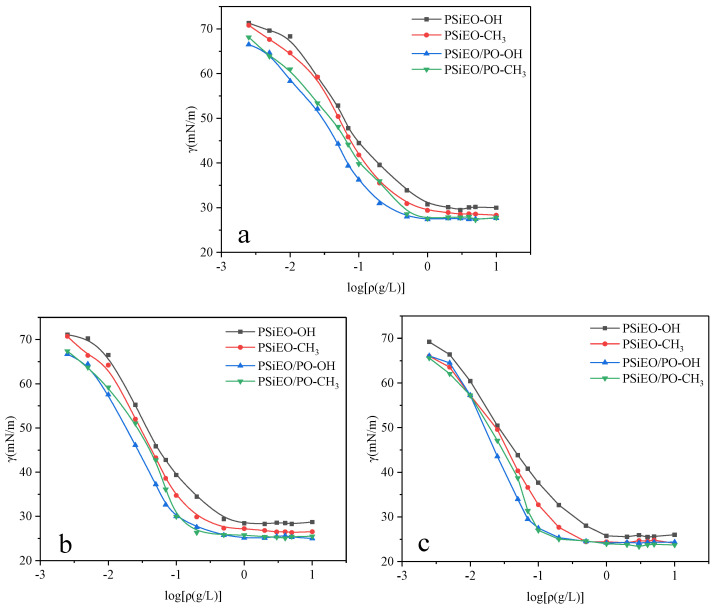
*γ* vs. log*C* curves of PSiEO/(PO)-OH(CH_3_) surfactants at (**a**) pH 3.0, (**b**) pH 7.0, and (**c**) pH 11.0.

The *γ* vs. log*C* curves of the surfactants at different solution pH are plotted in Figure 4. The surface tensions initially decreased gradually with increasing mass concentration, then dropped sharply, and finally reached a stable value. These changes were related to the adsorption of the surfactants at the gas–liquid interface. The polysiloxane blocks on the main chain were hydrophobic, causing them to extend into the air, whereas the polyether groups were hydrophilic, causing them to extend into the water [24]. At low concentrations, the surfactant molecules were sparsely distributed at the gas–liquid interface, resulting in a slow reduction of surface tension as the concentration increased. However, as the concentration of surfactants increased, the decrease in surface tension became more significant because of the increased adsorption density [25]. When the gas–liquid interface was saturated with surfactant molecules, the slope of the curve became almost constant. The transition and equilibrium points on the curve indicate the formation of micelles. Thus, The concentration corresponding to the intersection point of transition point and equilibrium point is referred to as the critical micelle concentration (CMC) [26].

The adsorption efficiency of surfactant is related to the interfacial area occupied by surfactant molecules. A larger minimum surface absorption area (*A*_min_) indicates a sparser surfactant arrangement on the surface of the solution, and vice versa [9]. *A*_min_ was calculated using Equation (2) [27]:

(2) $$A_{\min}=\frac{1}{N_{A}\Gamma_{\max}}\times 10^{23}$$

where *N*_A_ is Avogadro’s constant (6.02 × 10^23^).

Another useful parameter for characterizing the adsorption efficiency of surfactants is *pC*_20_, which represents the negative logarithm of the surfactant concentration required to reduce the surface tension by 20 mN m^−1^ (dyn m^−1^), as given by Equation (3). A higher *pC*_20_ value indicates a higher migration efficiency.

(3) $$pC_{20}=-\log C_{20}.$$

The CMC, *γ*_CMC_, *pC*_20_, *Γ*_max_, and *A*_min_ values of the PSiEO/(PO)-OH(CH_3_) surfactants are listed in Table 2.

The data in Table 2 show the effects of the propylene oxide (PO) chain and capping group on the CMC, *pC*_20_, and *A*_min_ values of the PSiEO/(PO)-OH(CH_3_) surfactants. The introduction of the PO chain, which increased the lipophilicity of the surfactants, led to a reduction in CMC and an increase in *pC*_20_. This indicates that surfactants with PO chains had a stronger ability to decrease the surface tension, which is consistent with the behavior of common quaternary ammonium Gemini surfactants and other surfactants [28]. Moreover, for the surfactants with different capping groups but the same hydrophilic chain, acetyl terminations offered more advantages in terms of reducing the surface tension of the solution in alkaline environments than hydroxyl terminations. In neutral and acidic environments, the capping group had little effect on the surface tension.

Comparing the *pC*_20_ values of surfactants with the same capping group at the same pH, the surfactants with PO chains were more efficient at reducing the surface tension. This indicates that surfactants with greater hydrophobicity exhibited greater *pC*_20_ values, which is consistent with the trends in surfactant behavior [21,24]. For the surfactants with pure ethylene oxide (EO) hydrophilic segments, those with acetyl terminations more efficiently decreased the surface tension. However, for the surfactants with EO/PO segments, the capping group had little effect on the surface tension.

The *A*_min_ values of the surfactants containing PO segments were smaller than those of the surfactants without PO segments. This was ascribed to differences in the flexibility of the surfactants with different chain lengths; the hydrophobic PO segments could bend more quickly, resulting in a reduction in *A*_min_ [29]. Table 2 shows that the *A*_min_ values of the surfactants were highest at pH 3.0 and lowest at pH 11.0. This is because the PSiEO/(PO)-OH(CH_3_) molecules existed as cations at pH 3.0, which led to electrostatic repulsion between and within the surfactant molecules. This electrostatic repulsion increased *A*_min_. By contrast, at pH 11.0, the PSiEO/(PO)-OH(CH_3_) molecules were nonionic, with no electrostatic repulsion between them, thereby decreasing *A*_min_.

### 3.4. Dynamic Surface Tension of PSiEO/(PO)-OH(CH_3_) Surfactants

Compared with the static surface tension, which measures the effectiveness of a surfactant, the dynamic surface tension reflects the efficiency of quick surface tension reduction, making it another important performance metric [30]. The dynamic surface tension is relevant for many interfacial applications, such as rapid wetting or foaming of textiles, coatings, and plastics, where static surface tension is not reached. In these cases, the time-dependent (dynamic) surface tension is more important than the equilibrium (static) surface tension.

**Figure 5:**
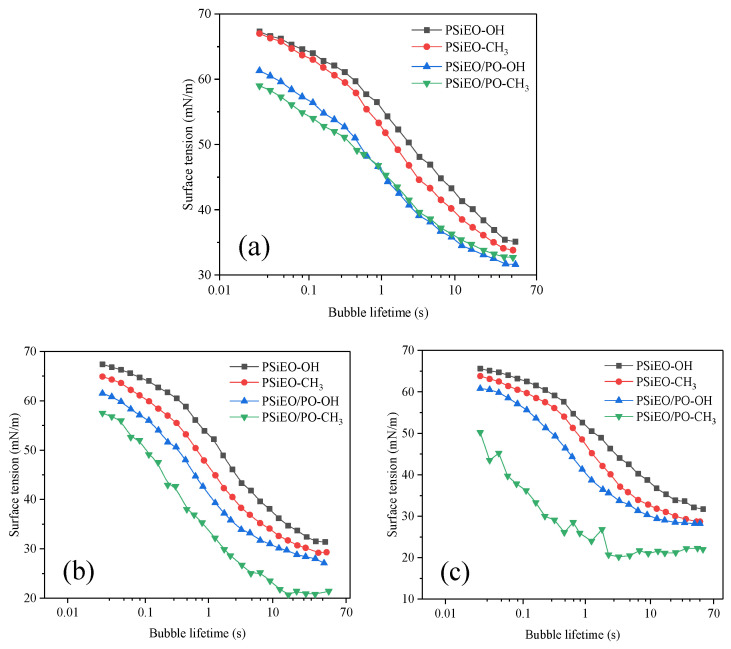
Dynamic surface tension curves of PSiEO/(PO)-OH(CH_3_) surfactants at (**a**) pH 3.0, (**b**) pH 7.0, and (**c**) pH 11.0.

The dynamic surface tension was tested using the maximum bubble pressure method [31] and calculated using a simplified form of the Young–Laplace equation (Equation (4)) [32]:

(4) $$\gamma_{t}=\frac{\Delta P_{\max}\times R_{\mathrm{cap}}}{2}$$

where *γ_t_* is the surface tension at time *t*, *R*_cap_ is the radius of the capillary, and Δ*P*_max_ is the maximum pressure drop. The dynamic surface tensions of the PSiEO/(PO)-OH(CH_3_) surfactants (concentration: 2 g L^−1^) were measured using a dynamic surface tension meter at 25 ± 0.2 °C. The relationship between the surface tension and the bubble lifetime is shown in Figure 5.

The total dynamic surface tension process can be split into four stages: induction, rapid surface tension decrease, medium equilibrium, and equilibrium [33]. The empirical equation in Equation (5) describes the change in dynamic surface tension:

(5) $$\frac{\gamma_{0}-\gamma_{t}}{\gamma_{t}-\gamma_{m}}={\left(\frac{t}{t^{*}}\right)}^{m}$$

where *γ*_0_ and *γ*_m_ are the surface tension of water and the solution at medium equilibrium, respectively, and t* and *m* are constants.

Taking the logarithms of both sides of Equation (5) gives

(6) $$\log\left(\frac{\gamma_{0}-\gamma_{t}}{\gamma_{t}-\gamma_{m}}\right)=m\left(\log t-\log t^{*}\right)$$

**Figure 6:**
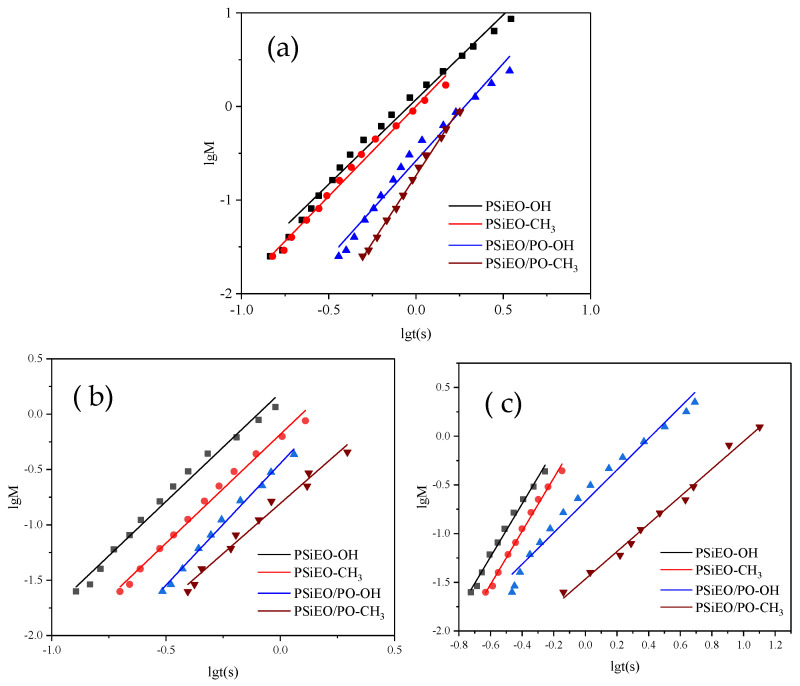
lg*M* vs. lgt(s) diagrams of PSiEO/(PO)-OH(CH_3_) surfactants at (**a**) pH 3.0, (**b)** pH 7.0, and (**c**) pH 11.0.

If (*γ*_0_ − *γ_t_*)/(*γ_t_* − *γ*_m_) = *M*, the values of t* and *n* can be calculated from the slope and intercept of the log*M* vs. log*t* plots, as shown in Figure 6. Further, the time at medium equilibrium (*t*_m_) and at the end of the induction interval (*t*_i_) and the reduction in surface tension at medium equilibrium (*R*_1/2_) are given by Equations (7)–(9):

(7) $$\log t_{m}=\log t^{*}+\frac{1}{m}$$

(8) $$\log t_{i}=\log t^{*}-\frac{1}{m}$$

(9) $$R_{1/2}=\frac{\gamma_{0}-\gamma_{m}}{2t^{*}}$$

As shown in Figure 6, the dynamic surface tensions of PSiEO-OH and PSiEO-CH_3_ were similar to those of PSiEO/PO-OH and PSiEO/PO-CH_3_ at pH 3.0, indicating that the capping group had little effect on the dynamic surface properties of the surfactants in the cationic/nonionic state. However, as the pH decreased, the end group had a greater influence on the dynamic performance of the surfactant. This was ascribed to the gradual removal of H^+^ by the quaternary ammonium salt. Notably, at pH 11.0, the dynamic surface tension of PSiEO/PO-CH_3_ was considerably unstable, possibly because of its poor hydrophilicity in the nonionic state, which hindered its migration to the interface by hydrophilicity. This indicates that this surfactant had a response behavior in the process of pH change from 7 to 11.

Table 3 shows the relationship between the dynamic surface tension parameters and the bubble lifetimes of the PSiEO/(PO)-OH(CH_3_) surfactants at different solution pH values. The *γ*_m_ values of PSiEO/(PO)-OH(CH_3_) in Table 2 followed a similar trend to the static surface tension values.

The *n* values reflect the diffusion of surface-active molecules from the solution to the newly generated surface in the initial adsorption stage. Smaller *n* values indicate less diffusion resistance and faster surfactant diffusion rates. As shown in Table 3, the *n* values of PSiEO-OH and PSiEO-CH_3_ increased with increasing pH, indicating that these surfactants were less likely to diffuse to the new interface as the solution pH increased. This is because increasing the solution pH decreased the charge carried by PSiEO-OH and PSiEO-CH_3_, and the interaction force between molecules, thereby increasing the mutual repulsion between molecules. In addition, it made the diffusion of molecules in solution more complex than that under acidic conditions. By contrast, PSiEO/PO-OH and PSiEO/PO-CH_3_ exhibited divergent interfacial behaviors as the *n* value changed at different pH values, which was ascribed to the hydrophobic effects of the PO segments in the hydrophilic groups. At pH 7.0–11.0, the PO-containing hydrophilic segments were more hydrophobic than the surfactants without EO. Consequently, the hydrophobicity of the molecules overcame the intermolecular attraction, making it easier for PSiEO/PO-OH and PSiEO/PO-CH_3_ to diffuse to the new interface in solution [34].

The t* value represents the adsorption time at which the surfactant reaches the surface of the solution at the end of the adsorption process. A smaller t* value indicates a larger adsorption barrier [35]. As shown in Table 3, at pH 3.0–7.0, PSiEO/PO-OH and PSiEO/PO-CH_3_ had larger t* values than the other two surfactants; at pH 11.0, the gap increased significantly. These results indicate that, as the hydrophobicity of the surfactant increased, the adsorption barrier of the surfactant at the end of the adsorption process decreased, especially in alkaline environments; when N lost its proton, PSiEO/(PO)-OH(CH_3_) lost its electrostatic repulsion and quaternary ammonium hydrogen bonds, considerably reducing the adsorption barrier.

The *R*_1/2_ value is the rate at which the surfactant decreases *γ*_0_ to *γ*_m_. A larger *R*_1/2_ value indicates a higher rate of surface tension reduction [32]. As shown in Table 3, all four surfactants exhibited the highest rate of surface tension reduction at pH 3.0. This is because the molecules were cationic under acidic conditions, resulting in high intermolecular repulsion. Therefore, the molecules were well-dispersed in the solution. However, as shown in Table 2, the surfactants had the strongest ability to reduce the surface tension at pH 11.0, indicating that these pH-responsive surfactants can be used for different purposes in different environments.

### 3.5. Thermodynamics of Micelles and Absorption of PSiEO/(PO)-OH(CH_3_) Surfactants

Micellar solutions formed by surfactant molecules are in thermodynamic equilibrium. Therefore, thermodynamic methods can be used to study the properties of micelles. Considering the amphiphilic nature of the surfactants, introducing hydrophobic groups increases the Gibbs free energy of the aqueous solution system. The Gibbs free energy of the system is reduced by two mechanisms [22]. First, when the hydrophobic groups of the surfactant molecules orient to the air side (i.e., adsorption), the Gibbs free energy is reduced. The Gibbs free energy change, enthalpy change, and entropy change of adsorption ($\Delta G_{\mathrm{ad}}^{\theta }$, $\Delta H_{\mathrm{ad}}^{\theta }$, and $\Delta S_{\mathrm{ad}}^{\theta }$, respectively; Equations (10)–(12)) [36] represent the changes in the thermodynamic properties during adsorption:

(10) $$\Delta G_{\mathrm{ad}}^{\theta }=RT\ln X_{CMC}-\left(\gamma_{0}-\gamma_{CMC}\right)N_{A}A_{\min}$$

(11) $$\Delta H_{\mathrm{ad}}^{\theta }=-T^{2}\frac{\partial \left(\Delta G_{\mathrm{ad}}^{\theta }/T\right)}{\partial T}$$

(12) $$\Delta S_{\mathrm{ad}}^{\theta }=\frac{\left(\Delta H_{\mathrm{ad}}^{\theta }-\Delta G_{\mathrm{ad}}^{\theta }\right)}{T}$$

where *X_CMC_* is the CMC expressed as a molar fraction and $\partial \left(\Delta G_{\mathrm{ad}}^{\theta }/T\right)/\partial T$ is the slope of the *T* vs. $\Delta G_{\mathrm{ad}}^{\theta }/T$ curve.

Second, when the surfactants aggregate to form micelles, their hydrophobic groups situate toward the interior of the micelles. This process is known as micellization. The thermodynamic parameters $\Delta G_{\mathrm{mic}}^{\theta }$, $\Delta H_{\mathrm{mic}}^{\theta }$, and $\Delta S_{\mathrm{mic}}^{\theta }$ represent the Gibbs free energy change, enthalpy change, and entropy change of micelle formation, respectively (Equations (13)–(15)):

(13) $$\Delta G_{\mathrm{mic}}^{\theta }=RT\ln X_{CMC}$$

(14) $$\Delta H_{\mathrm{mic}}^{\theta }=-T^{2}\frac{\partial \left(\Delta G_{\mathrm{mic}}^{\theta }/T\right)}{\partial T}$$

(15) $$\Delta S_{\mathrm{mic}}^{\theta }=\frac{\left(\Delta H_{\mathrm{mic}}^{\theta }-\Delta G_{\mathrm{mic}}^{\theta }\right)}{T}$$

The $\Delta G_{\mathrm{ad}}^{\theta }$ and $\Delta G_{\mathrm{mic}}^{\theta }$ values of the PSiEO/(PO)-OH(CH_3_) surfactants were calculated based on the measured *X*_CMC_ and *γ*_CMC_ values using Equation (10) and Equation (13), respectively; $\Delta H_{\mathrm{ad}}^{\theta }$ and $\Delta H_{\mathrm{mic}}^{\theta }$ were calculated using Equation (11) and Equation (14), respectively; and $\Delta S_{\mathrm{ad}}^{\theta }$ and $\Delta S_{\mathrm{mic}}^{\theta }$ were calculated using Equation (12) and Equation (15), respectively.

As shown in Table 4, all the $\Delta G_{\mathrm{ad}}^{\theta }$ and $\Delta G_{\mathrm{mic}}^{\theta }$ values were negative, indicating that the adsorption and micellization of these surfactants occurred spontaneously [37,38]. The negative values of $\Delta G_{\mathrm{ad}}^{\theta }$ and $\Delta G_{\mathrm{mic}}^{\theta }$ were mainly determined by the large values of $\Delta S_{\mathrm{ad}}^{\theta }$ and $\Delta S_{\mathrm{mic}}^{\theta }$; thus, the interfacial adsorption and micellization processes were dominated by an increase in entropy. The driving force for these processes came from the transfer of the hydrophobic group of the surfactant to the interface or micelle core.

$\Delta G_{\mathrm{ad}}^{\theta }$ and $\Delta G_{\mathrm{mic}}^{\theta }\;$were calculated using Equation (10) and Equation (13), respectively, based on surface tension and CMC data; $\Delta H_{\mathrm{ad}}^{\theta }$ and $\Delta H_{\mathrm{mic}}^{\theta }$ were derived from the temperature dependence of surface tension (25–45 °C) via van ’t Hoff analysis; $\Delta S_{\mathrm{ad}}^{\theta }$ and $\Delta S_{\mathrm{mic}}^{\theta }$ were calculated using Equation (15).

For the PSiEO/(PO)-OH(CH_3_) surfactants, the introduction of hydrophobic PO groups and acetyl end-capping increased $\Delta S_{\mathrm{ad}}^{\theta }$ and $\Delta S_{\mathrm{mic}}^{\theta }$ and decreased $\Delta H_{\mathrm{ad}}^{\theta }$ and $\Delta H_{\mathrm{mic}}^{\theta }$. However, owing to the large change in $\Delta S_{\mathrm{ad}}^{\theta }$ and $\Delta S_{\mathrm{mic}}^{\theta }$, the absolute values of $\Delta G_{\mathrm{ad}}^{\theta }$ and $\Delta G_{\mathrm{mic}}^{\theta }$ increased By contrast, introducing hydrophilic groups increased $\Delta H_{\mathrm{ad}}^{\theta }$, $\Delta H_{\mathrm{mic}}^{\theta }$, $\Delta S_{\mathrm{ad}}^{\theta }$, and $\Delta S_{\mathrm{mic}}^{\theta }$, leading to a slight increase in the absolute values of $\Delta G_{\mathrm{ad}}^{\theta }$ and $\Delta G_{\mathrm{mic}}^{\theta }$. The enthalpy change indicates whether the adsorption or micellization process is dominated by bond formation ($\Delta H_{\mathrm{ad}}^{\theta }$ or $\Delta H_{\mathrm{mic}}^{\theta }$ < 0) or bond cleavage ($\Delta H_{\mathrm{ad}}^{\theta }$ or $\Delta H_{\mathrm{mic}}^{\theta }$ > 0). During the process of adsorption and micellization, the increase in enthalpy may be because the EO group needs to be partially dehydrated during interfacial migration or micellization. The dehydration process is endothermic, and the extent of dehydration increases with the number of EO units. An explanation for the increase in entropy during adsorption and micellization in aqueous solutions has been proposed [39]: when the surfactant is transferred from the aqueous phase to the interface or micelle state, the internal degrees of freedom of the hydrophobic segment increase compared with those in the aqueous environment. The thermodynamic parameters of the PSiEO/(PO)-OH(CH_3_) surfactants at different solution pH values indicate that, at pH 11, owing to the absence of electrostatic interaction, the chain segments had more degrees of freedom, so the entropy increase was higher. By contrast, at pH 3, the entropy increase was lower owing to electrostatic repulsion. Furthermore, the surfactant enthalpy decreased with increasing pH owing to an increase in the number of hydrophobic groups during the dehydration process.

Overall, the thermodynamic parameters indicated that the micellization and adsorption processes were endothermic, spontaneous, and mainly driven by entropy; increasing the pH and number of hydrophobic groups hindered these processes. Notably, while negative $\Delta G_{\mathrm{mic}}^{\theta }$ values confirm the spontaneity of micellization, this process is thermodynamically viable only when the surfactant concentration exceeds the critical micelle concentration (CMC). Below the CMC, monomers dominate in solution, and micelle formation is negligible despite the favorable $\Delta G_{\mathrm{mic}}^{\theta }$. The CMC threshold reflects the balance between the energy gain from hydrophobic interactions (driving micellization) and the entropic penalty of monomer organization into ordered micelles. This is consistent with our observation that surfactants with lower CMC values as PSiEO/PO-CH_3_ exhibited more negative $\Delta G_{\mathrm{mic}}^{\theta }$ (Table 4), as enhanced hydrophobicity reduces the entropic cost of micellization.

### 3.6. Aggregation Behavior of PSiEO/(PO)-OH(CH_3_) Surfactants

**Figure 7:**
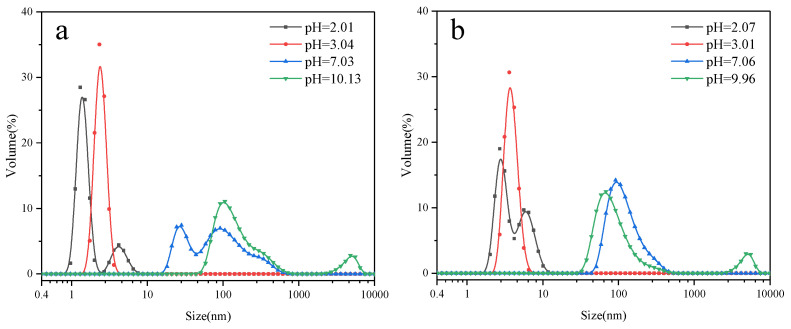

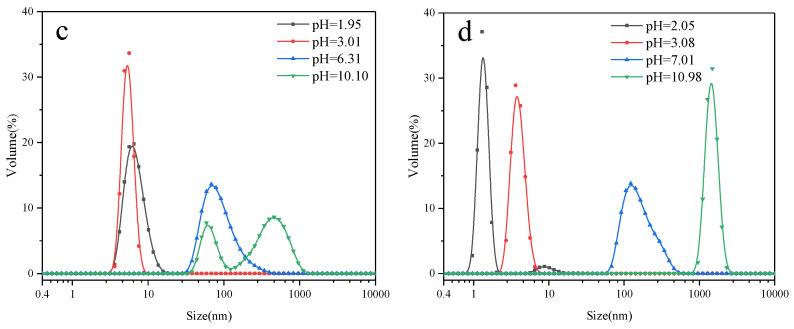
DLS of PSiEO/(PO)-OH(CH_3_) surfactant solutions at different pH values: (**a**) PSiEO-OH, (**b**) PSiEO-CH_3_, (**c**) PSiEO/PO-OH, and (**d**) PSiEO/PO-CH_3_.

The aggregation behavior of the PSiEO/(PO)-OH(CH_3_) surfactants was investigated under varying pH conditions through dynamic light scattering (DLS) and visual observations. As shown in Figure 7, the hydrodynamic diameter of surfactant aggregates increased significantly with rising pH, from 1–10 nm at pH 3 to >100 nm at pH 11. This trend directly correlated with the zeta potential measurements (Figure 3), where the absolute zeta potential decreased from >30 mV (pH 3) to near-neutral values (pH 11). The reduction in electrostatic repulsion at higher pH (e.g., pH 8–11) facilitated molecular aggregation, as evidenced by the DLS data. Under acidic conditions (pH 3), the protonated tertiary amine groups imparted a strong cationic charge, stabilizing the colloidal dispersion via electrostatic repulsion. In contrast, deprotonation at alkaline pH (≥8) eliminated this charge screening, allowing hydrophobic interactions between PO segments and acetyl capping groups to dominate, thereby promoting aggregation.

The synergy between zeta potential and DLS results clarifies the pH-dependent aggregation mechanism:

- At pH 3–5 (zeta potential > 30 mV): High electrostatic repulsion prevented aggregation (1–10 nm);
- At pH 6–8 (zeta potential 10–30 mV): Partial charge neutralization initiated micelle growth (10–100 nm);
- At pH > 9 (zeta potential < 10 mV): Electrostatic shielding allowed hydrophobic-driven aggregation (>100 nm).

Structural variations significantly influenced the aggregation thresholds. For example, PSiEO/PO-CH_3_ exhibited the largest aggregation threshold (~1000 nm at pH 11) due to its combined hydrophobic PO segments and acetyl capping, which enhanced intermolecular association (Figure S7). This aligned with its lower zeta potential (−5 mV at pH 11), indicating minimal repulsion between particles. In comparison, the hydroxyl-terminated surfactant (PSiEO-OH) showed smaller aggregates (500 nm at pH 11), as its polar terminal groups partially counterbalanced the hydrophobic driving force. These observations aligned with the thermodynamic parameters (Table 4), where surfactants with higher hydrophobicity (PSiEO/PO-CH_3_) exhibited more negative $\Delta G_{\mathrm{mic}}^{\theta }$ values, confirming their propensity for spontaneous micelle growth under alkaline conditions. Such behavior is critical for applications requiring pH-triggered aggregation, such as controlled drug release or self-healing coatings.

## 4. Conclusions

In this study, we synthesized a series of polyether- and tertiary amine-modified silicone surfactants and characterized their structures by various methods, such as ^1^H NMR and FTIR spectroscopy. Static and dynamic surface tension, zeta potential, and DLS measurements were also conducted at different pH values. The PSiEO/(PO)-OH(CH_3_) surfactants had different surface performances over a wide pH range. At low pH, PSiEO/(PO)-OH(CH_3_) changed from a nonionic surfactant to a cationic/nonionic one. Upon increasing the content of hydrophilic/hydrophobic groups, the charged core of the surfactant increased, the static surface tension increased, and the diffusion rate in the solvent increased. The thermodynamic parameters indicated that the micellization and adsorption processes of these surfactants were endothermic, spontaneous, and mainly driven by entropy; increasing the pH and number of hydrophobic groups hindered these processes. The aggregation behavior also changed significantly under acidic, neutral, and basic aqueous conditions. Among the surfactants, PSiEO/PO-CH_3_ had the lowest CMC and static surface tension; the highest *n* and t* values; and the lowest *R*_1/2_ value at pH 3, 7, and 11. Consequently, it exhibited the best migration efficiency. This was ascribed to the PO segments and acetyl terminations. The PSiEO/PO-CH_3_ surfactant also had the lowest $\Delta G_{\mathrm{ad}/\mathrm{mic}}^{\theta }$ and $\Delta H_{\mathrm{ad}/\mathrm{mic}}^{\theta }$ values and largest $\Delta S_{\mathrm{ad}/\mathrm{mic}}^{\theta }$ value. The thermodynamic parameters were consistent with the static and dynamic surface tension results. This surfactant also exhibited better pH correspondence performance. Owing to these surface properties, these pH stimuli-responsive surfactants offer potential for application in fields such as drug encapsulation and release, self-cleaning coatings, pH transducers, and other pH-responsive applications.

## Figures and Tables

**Table 1:** p*K*_a_ values of PSiEO/(PO)-OH(CH_3_) surfactants (concentration: 2 g L^−1^) at 25 °C.

Surfactant	p*K*_a_
PSiEO-OH	4.40/8.55
PSiEO-CH_3_	4.19/8.60
PSiEO/PO-CH_3_	4.35/8.07
PSiEO/PO-OH	4.30/8.19

**Table 2:** Surface properties of PSiEO/(PO)-OH(CH_3_) surfactants at 25 °C.

Surfactant	pH	CMC [g L^−1^]	*γ*_CMC_ [mN m^−1^]	*pC*_20_	*Γ*_max_ [μmoL m^−2^]	*A*_min_ [nm^2^]
PSiEO-OH	3.0	0.48	29.83	1.28	1.71	0.97
	7.0	0.38	28.22	1.47	2.00	0.83
	11.0	0.33	25.56	1.66	2.21	0.75
PSiEO-CH_3_	3.0	0.60	29.20	1.36	1.34	1.23
	7.0	0.33	27.44	1.60	1.66	1.00
	11.0	0.24	24.50	1.74	2.09	0.79
PSiEO/PO-OH	3.0	0.37	27.80	1.63	1.87	0.89
	7.0	0.14	25.26	1.81	2.47	0.67
	11.0	0.11	24.39	1.85	2.69	0.62
PSiEO/PO-CH_3_	3.0	0.42	28.06	1.52	1.64	1.01
	7.0	0.13	27.57	1.65	2.70	0.62
	11.0	0.12	22.95	1.80	3.41	0.49

**Table 3:** Calculated dynamic surface tension parameters.

pH	Surfactant	*γ*_m_ [mN m^−1^]	*n*	t* [s]	*R*_1/2_ [mN m^−1^ s^−1^]
3.0	PSiEO-OH	35.1	1.81	0.72	25.6
	PSiEO-CH_3_	33.8	1.92	1.00	19.1
	PSiEO/PO-OH	31.6	2.08	1.56	12.9
	PSiEO/PO-CH_3_	32.7	2.87	1.81	10.9
7.0	PSiEO-OH	31.4	1.96	0.80	25.4
	PSiEO-CH_3_	29.2	1.97	1.24	17.3
	PSiEO/PO-OH	27.1	2.20	1.90	11.8
	PSiEO/PO-CH_3_	20.7	1.80	2.82	9.1
11.0	PSiEO-OH	31.7	2.73	0.91	22.1
	PSiEO-CH_3_	28.7	2.73	1.39	15.6
	PSiEO/PO-OH	28.2	1.61	2.60	8.4
	PSiEO/PO-CH_3_	20.2	1.41	10.90	2.4

**Table 4:** Thermodynamic parameters of surface adsorption and micellization of PSiEO/(PO)-OH(CH_3_) surfactants.

Surfactant	pH	$\Delta G_{ad}^{\theta }$ [kJ mol^−1^]	$\Delta H_{ad}^{\theta }$ [kJ mol^−1^]	$\Delta S_{ad}^{\theta }$ [J mol^−1^ K^−1^]	$\Delta G_{mic}^{\theta }$ [kJ mol^−1^]	$\Delta H_{mic}^{\theta }$ [kJ mol^−1^]	$\Delta S_{mic}^{\theta }$ [J mol^−1^ K^−1^]
PSiEO-OH	3.0	−23.61	13.72	104.36	−21.15	9.96	125.20
	7.0	−26.42	7.90	106.83	−24.23	7.62	115.11
	11.0	−28.68	4.46	108.18	−26.58	5.67	111.15
PSiEO-CH_3_	3.0	−27.27	11.51	107.21	−24.10	7.87	130.06
	7.0	−28.26	6.95	108.80	−25.58	6.86	118.10
	11.0	−30.63	3.56	111.32	−28.37	4.82	114.65
PSiEO/PO-OH	3.0	−26.66	9.87	109.70	−24.30	8.41	122.55
	7.0	−29.59	6.48	113.96	−27.70	6.27	120.99
	11.0	−33.08	3.49	117.31	−31.30	3.67	122.67
PSiEO/PO-CH_3_	3.0	−27.65	8.37	112.76	−24.98	8.64	120.83
	7.0	−30.55	5.95	115.07	−28.89	5.42	122.41
	11.0	−34.53	3.20	119.62	−33.09	2.58	126.56

## Data Availability

The data presented in this study are openly available in the article.

## Supplementary Materials

The following supporting information can be downloaded at: https://www.mdpi.com/article/10.3390/polym17091204/s1, Figure S1: ^1^H-NMR spectrum of PSiEO-OH; Figure S2: ^1^H-NMR spectrum of PSiEO-CH_3_; Figure S3: ^1^H-NMR spectrum of PSiEO/PO-OH; Figure S4: ^1^H-NMR spectrum of PSiEO/PO-CH_3_; Figure S5: ^1^H NMR spectra of PSiEO/PO-OH at different solution pD; Figure S6: ^1^H NMR spectra of PSiEO/PO-OH in D_2_O/NaOD (pD 11) and CDCl_3_; Figure S7: Photograph of 0.5 g L^−1^ PSiEO/PO-CH_3_ solutions at different pH.
