# Supplementary material for: Polyether- and Tertiary Amine-Modified Silicone Surfactants: Synthesis and Surface Performance Across pH Ranges
Source: Polymers (Basel). 2025 Apr 28;17(9):1204. doi: 10.3390/polym17091204 (PMC12073686; doi:10.3390/polym17091204)
Supplement: Supplementary file 1 [file polymers-17-01204-s001.zip › polymers-3566963-supplementary.pdf]

# Supporting Information

**Polyether- and Tertiary Amine-Modified Silicone Surfactants: Synthesis and Surface Performance Across pH Ranges**

*Yi Guo, Qiansong Wu, and Cheng Yao**

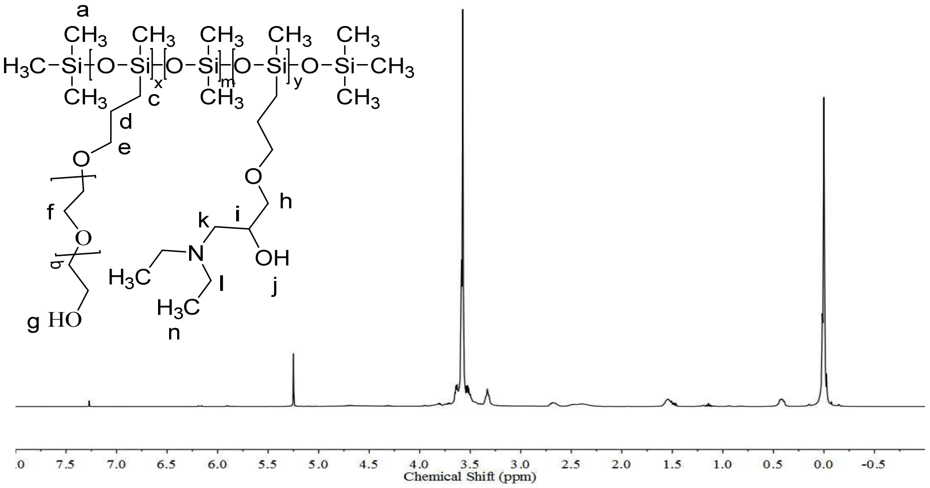


**Figure S1.** $^{1}$H-NMR spectrum of PSiEO-OH. $^{1}$H NMR ($CDCl_3$, ppm): 0.075 (a H), 0.43 (c H), 1.14 (d H), 1.15 (n H), 2.41 (l H), 2.68 (k H), 3.33 (e H), 3.53 (f H), 3.58 (h H), 3.64 (i H), 5.25 (g H), 5.25 (j H), 7.27 ($CDCl_3$).

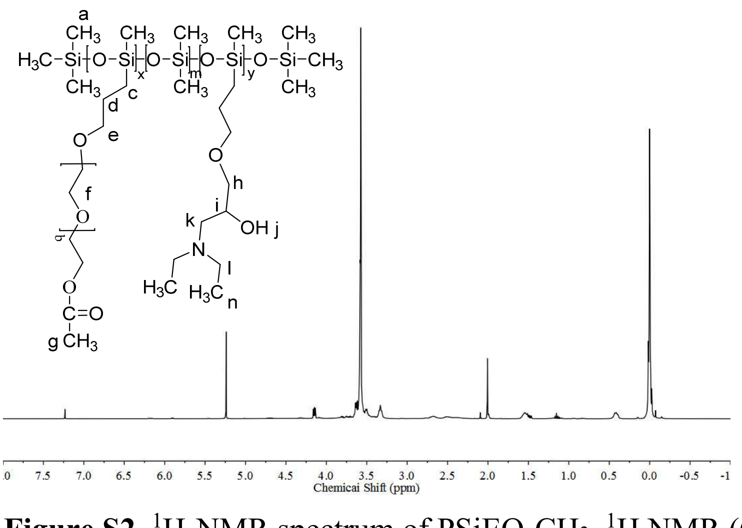


**Figure S2.** $^{1}$H-NMR spectrum of PSiEO-$CH_3$. $^{1}$H NMR ($CDCl_3$, ppm): 0.075 (a H), 0.42 (c H), 1.15 (d, n H), 2.01 (g H), 2.49 (l H), 2.68 (k H), 3.33 (e H), 3.51 (h H), 3.56 (f H), 3.71 (i H), 5.25 (j H), 7.23 ($CDCl_3$).

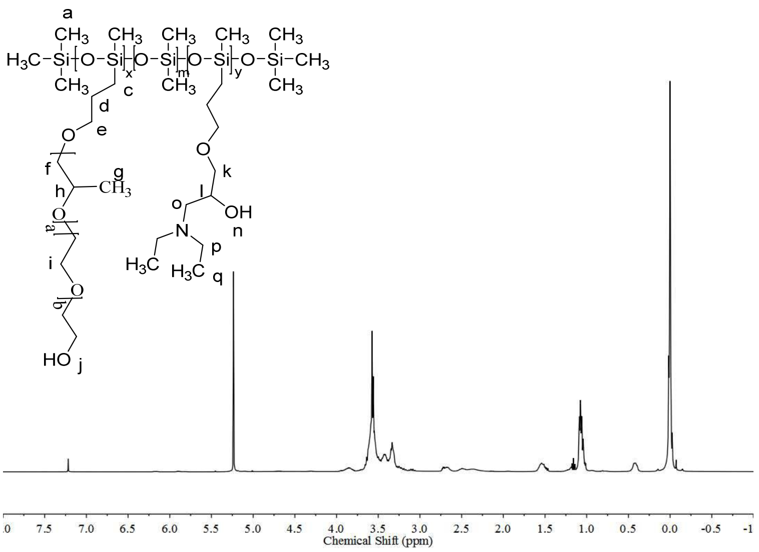


**Figure S3.** $^{1}$H-NMR spectrum of PSiEO/PO-OH. $^{1}$H NMR ($CDCl_3$, ppm): 0.075 (a H), 0.42 (c H), 1.08 (g H), 1.15 (d, q H), 2.41 (o H), 2.71 (p H), 3.33 (h H), 3.53 (e, f, i H), 3.63 (k H), 3.82 (l H), 5.24 (j H), 5.24 (n H), 7.23 ($CDCl_3$).

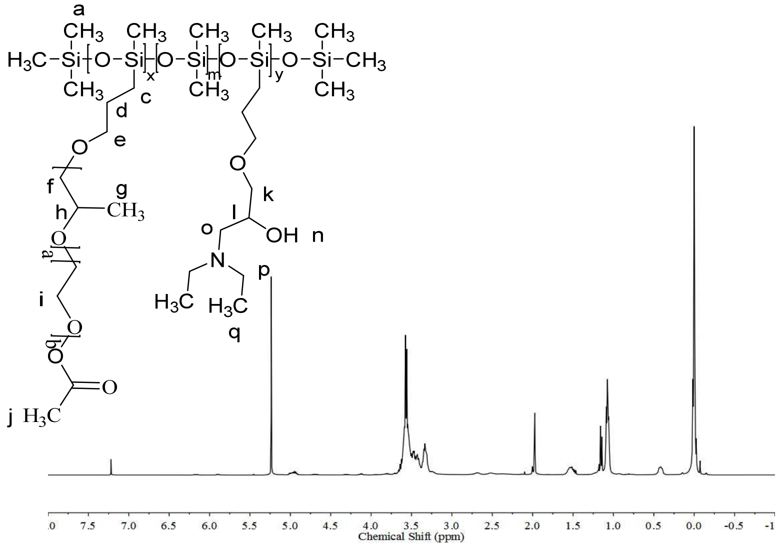


**Figure S4.** $^{1}$H-NMR spectrum of PSiEO/PO-$CH_3$. $^{1}$H NMR ($CDCl_3$, ppm): 0.075 (a H), 0.43 (c H), 1.08 (g H), 1.16 (d, q H), 1.97 (j H), 2.52 (o H), 2.71 (p H), 3.33 (h H), 3.58 (e, f, i H), 3.63 (k H), 3.65 (l H), 5.24 (n H), 7.23 ($CDCl_3$).

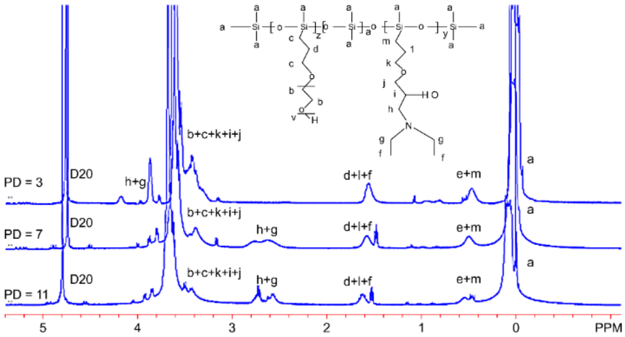


**Figure S5.** $^{1}$H NMR spectra of PSiEO/PO-OH at different solution pD. $^{1}$H NMR (pD 11, $D_2O$/NaOD, ppm): 0.075 (a H), 0.43 (e+m H), 1.47–1.52 (d+l+f H), 3.11–3.75 (b+c+i+j+k H), 3.84 (h+g H), 4.7 ($D_2O$). $^{1}$H NMR (pD 7, $D_2O$, ppm): 0.075 (a H), 0.43 (e+m H), 1.47–1.52 (d+l+f H), 2.59 (h+g H), 3.11–3.75 (b+c+i+j+k H), 4.7 ($D_2O$). $^{1}$H NMR (pD 3, $D_2O$/DCl, ppm): 0.075 (a H), 0.43 (e+m H), 1.47–1.52 (d+l+f H), 2.59 (h+g H), 3.11–3.75 (b+c+i+j+k H), 4.7 ($D_2O$).

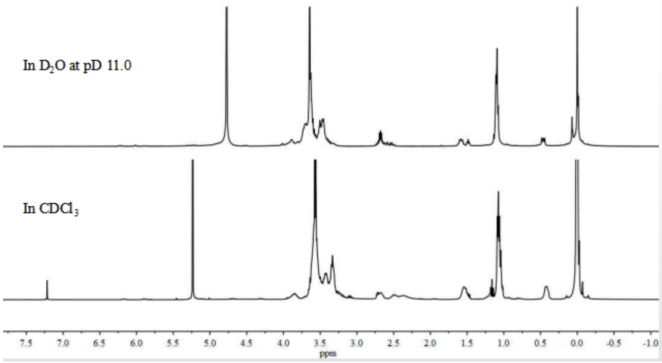


**Figure S6.** $^{1}$H NMR spectra of PSiEO/PO-OH in $D_2O$/NaOD (pD 11) and $CDCl_3$.

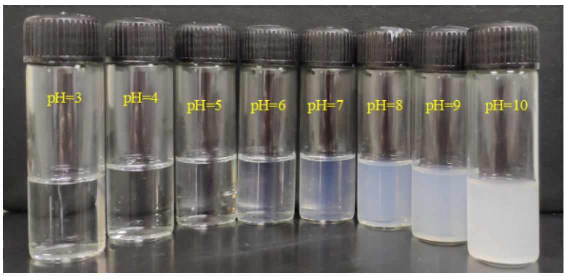


**Figure S7.** Photograph of 0.5 g $L^{-1}$ PSiEO/PO-$CH_3$ solutions at different pH .
